# Challenges and prospects in using biotechnological interventions in *O. glaberrima*, an African cultivated rice

**DOI:** 10.1080/21645698.2022.2149212

**Published:** 2022-12-01

**Authors:** Gideon Sadikiel Mmbando

**Affiliations:** Department of Biology, College of Natural and Mathematical Sciences, University of Dodoma (Udom), Dodoma, Tanzania

**Keywords:** Gene editing, genetic engineering, *O. glaberrima*, regeneration, transformation

## Abstract

Africa has the world’s fastest rate of population expansion, making it vulnerable to food shortages. Africa cultivates two types of rice (Asian rice; *Oryza sativa* and African rice; *Oryza glaberrima*). Native African rice called *O. glaberrima* has some intriguing characteristics, including resistance to several biotic and abiotic regional restrictions in Africa. However, *O. glaberrima* is solely employed as a tool to increase the production of *O. sativa*, which cannot grow in Africa, due to its low yield, lodging, grain breaking, and poor tissue culture ability. Enhancing breeding efforts for *O. glaberrima* is therefore critically important. The protocols for transformation and regeneration, however, are mostly for *O. sativa* and not *O. glaberrima*. This study examines the present problems with transformation and regeneration for African rice species as well as potential solutions for using modern breeding methods in *O. glaberrima*.

## Introduction

The phrase “Rice is life” captures the importance of this ancient grain to people as a main source of nutrition as well as spiritual and cultural nourishment, not just in Asia^1^ but also in Africa. More than half of the world’s population is fed by rice, making it the most significant cereal crop.^2^ In addition, rice has developed into a model monocot system for genetic and functional genomic research.^3^ Only two of the 23 species in the genus *Oryza* have been grown for food: *Oryza sativa* and *Oryza glaberrima*.^4^ *O. glaberrima* Steud. was domesticated in West Africa,^5^ whereas *O. sativa* was in Asia.^6^ Both species are grown in Africa, and varieties of *O. sativa* were brought to West Africa as a result of trade between Africa and India.^7^ *O. glaberrima*’s yield is thought to be lower than *O. sativa*’s, probably as a result of many features such as lodging and grain breaking,^8^ and as a result, *O. sativa* has continued to take over its growing regions.^8^ *O. glaberrima*, however, is still grown in Africa today^9^ and is preferred by local farmers over *O. sativa* for some reasons, including resilience to a variety of regional restrictions, taste, hardiness, and resistance to many biotic and abiotic stresses.^10^ It’s been reported that farmers abandoned better *O. sativa* cultivars to resume the cultivation of *O. glaberrima*. For instance, *O. glaberrima* is currently grown in a deep water location at Banfora in Burkina Faso, despite the government having already introduced a deep water variety from Indonesia^11^; demonstrating the strong resilience of *O. glaberrima* to such severe environments, including deep water areas. However, there is a lack of an appropriate breeding and genetic development program to benefit from *O. glaberrima*’s advantages. This has kept the unwanted traits from being removed from this species while allowing the beneficial traits to be transferred to *O. sativa*, which is not an African native and cannot survive there.

Although it has been reported that the New Rice for Africa (NERICA) varieties combine some ability to develop well in the challenging environment of *O. glaberrima* as well as the high-yielding potential of *O. sativa*^5,12,13^; they still lack resistance mechanisms to some local constraints, including weeds, in comparison to *O. glaberrima* variety.^14^ As a result, Africa Rice continues to look for *O. glaberrima* lines for crucial characteristics in rainfed rice in Sub-Saharan Africa (SSA). *O. glaberrima* has, however, received far less scientific attention, particularly in the areas of genetic breeding and biotechnology.^10^ *O. glaberrima*’s undesirable traits, such as grain breaking, lodging,^5,15,16^ high ultraviolet-B (UVB) sensitivity,^17^ and limited yield potential, may have led to its usage primarily as a genetic resource to enhance *O. sativa*.^18^ To fully utilize current breeding techniques in enhancing the yield and characteristics of this species for the people of Africa and the rest of the world, it is crucial to produce appropriate transformation and regeneration of *O. glaberrima* local varieties.

This review covers the current status, challenges, and potential solutions to promote transformation and regeneration with an emphasis on African rice. This study urges the creation of a transformation and regeneration protocol for *O. glaberrima* immediately. This study also made several suggestions for how the most cutting-edge genome editing techniques, such as clustered regularly interspaced short palindromic repeats (CRISPR)-associated endonuclease Cas9 (CRISPR/Cas9), might be used to improve *O. glaberrima*’s yield and eliminate its undesirable characteristics. This is because *O. glaberrima* is an elite line that has adapted to the African environment. Such knowledge is crucial for the biotechnological advancement of African rice, which will hasten the continent’s green revolution by supplying dream rice with all the nutritional benefits that developing nations most want.

## 1. Importance of *O. Glaberrima* to Africa

Due to climate fluctuation, rice farming in Africa is unstable and susceptible to numerous environmental challenges, including drought and submergence.^19^ Therefore, weak features in native and local rice cultivars that are already acclimated to other pressures in SSA should be genetically improved to create efficiently stable and sustainable agricultural systems in Africa. *O. glaberrima* is thought to have evolved tolerance to the majority of abiotic and biotic stressors for rice farming in SSA^5,20^ since it has thrived in Africa with little intervention from humans. *O. glaberrima*, for instance, is recognized as a source of resistance to a variety of native abiotic challenges, including low phosphorus availability in acid soils,^21^ submergence,^22,23^ drought,^5,20,24,25^ iron toxicity,^26^ and biotic challenges like nematodes,^27–30^ African rice gall midges,^31,32^ weeds,^33–35^ and the rice yellow mottle virus^36,37^ *O. glaberrima* is employed in traditional ritual rites to satisfy the souls of the ancestors, as in the communities of the Danyi plateau in Togo and the Casamance region in southern Senegal.^38^ This is in addition to its resistance to many geographical restrictions in Africa. The genetic diversity of this species may also be an important breeding resource. For instance, increasing the amount of certain micronutrients^10,39–41^ and increasing the amount of protein in grains.^42–44^ As a result, genetically enhancing *O. glaberrima*’s poor features will have the added benefit of all these advantageous traits acting as adaptation mechanisms, which will aid the organism’s ability to thrive in the stress-prone SSA region.

*O. glaberrima* has a great degree of adaptation to local farming in Africa, which may explain why it is still grown in some regions of West Africa today.^9^ It is a big surprise that rice researchers have neglected *O. glaberrima* breeding initiatives for many years in favor of focusing on raising the productivity of Asian rice varieties to increase food supply in both Asia and Africa, despite all the advantages described above. Very few studies have attempted to *increase O. glaberrima* yield by fusing the positive characteristics of *O. glaberrima* and *O. sativa*, as demonstrated in NERICA.^5,12,13^ What is surprising is that *O. glaberrima* has been overlooked even by African researchers studying rice and primarily uses it as a source to raise the yield of *O. sativa*. The lack and poor regeneration capacity of this species in comparison to *O. sativa* could be one explanation for this^45,46^ due to the paucity of data on *O. glaberrima*’s callus induction and plantlet regeneration.^47^ The lack of suitable regeneration media may be the reason why the majority of rice researchers have given up on breeding and genetic enhancement programs for *O. glaberrima*, in addition to the sterility barrier between the two domesticated species.^48,49^ Similar to what has already been done on Asian rice varieties, rice researchers urgently need to create the ideal conditions for transformation and regeneration media for native African rice varieties. Future research should investigate this topic. The recent development in gene editing and modification allows us to artificially disrupt a single gene responsible for the reproductive barrier,^50,51^ allowing us to avoid the hybrid sterility of *O. glaberrima* and *O. sativa* and thereby facilitate interspecific hybridization for breeding programs of the two species.

Alternatively, *O. glaberrima*’s unfavorable traits may have led to its use primarily as a genetic resource to enhance *O. sativa*.^8^ This shouldn’t be a problem today, though, as recent developments in genome editing technologies, like the CRISPR/Cas 9 system,^52–54^ may make it possible to eliminate undesirable traits in *O. glaberrima* and even hasten the process of domestication.^55^ The best way to improve *O. glaberrima*, for instance, maybe to use CRISPR/Cas9 technology to edit and modify the genes responsible for traits like grain shattering and lodging resistance. These traits are crucial in the improvement of *O. glaberrima*.^56^ A different approach is to look for *O. glaberrima* lines that are resilient to grain lodging and shattering. Although lines that are resistant to lodging have not yet been identified,^10^ this has already begun. It is significant to note that proper transformation and regeneration protocols, which are thought to be crucial steps during the generation of transgenic plants,^57^ are required for the success of these gene editing technologies, including CRISPR/Cas9.

## 2. Challenges in Improving *O. Glaberrima* through Hybridization

Genetic advancements in cereals, particularly those of economically significant crops like rice, are considered to be a promising solution to the issue of a food shortage.^58^ However, the establishment of good callus induction and subsequent plant regeneration are the key steps^59,60^ before utilizing any genetic improvement program. It is well known that the accessibility of gene transfer, which is inappropriate for plant regenerations, makes it difficult for many transgenic species to recover.^57^ During somatic embryogenesis, cell division and differentiation are significantly influenced by plant growth regulators.^61,62^ Therefore, embryogenic callus is essential for successful regeneration, and *O. glaberrima* may have less regeneration potential than *O. sativa* because it forms fewer embryogenic calluses. Even under different hormonal concentrations, Mmbando et al. showed that MS media^63^ rather than N6^64^ was the most effective for inducing calluses in the majority of *O. glaberrima* varieties. On the other hand, the control Asian rice cultivar Nipponbare appears to react strongly to N6 media. The variation in callus induction between Nipponbare and African rice varieties is brought on by differences in the nutrient media composition and genotype, which is one of the major factors for tissue culture.^65^ Future research should test various hormonal concentration gradients using the best MS media for *O. glaberrima* suggested for cultivar TOG12380.^66^

The majority of cultivars of *O. glaberrima* also showed cell necrosis, though the cause is still unknown, which prevents effective regeneration.^66^ This report resembles one that was done previously on *indica* rice (*O. sativa*) ^67^ Numerous plant species that struggle with *in vitro* culture have been found to exhibit callus browning, which reduced their ability to grow and regenerate and even caused their death.^68^ Similar to this, Mmbando et al. showed that only 1 of the 5 *O. glaberrima* tested cultivars was regenerated by Murashinge and Skoog (MS)^61^ using media from Brisibe et al.^63^ while the others formed tiny brown calli during the callus induction stage.^66^ Maltose has been shown to cause less browning than sucrose when added to culture media.^69^ Although there are numerous protocols for embryogenic calli-mediated plant regeneration in *indica* rice among *O. sativa* varieties, the majority of them are genotype-dependent, and the universal medium that can be adapted to a wide range of *indic*a rice genotypes has not yet been developed.^70^ The majority of callus induction protocols created for *O. sativa*, unless modified do not function well in *O. glaberrima* and frequently cause the callus to brown, necrosis, and grow poorly, all of which ultimately harm callus regeneration. Despite *O. glaberrima* tissue culture can be produced and increased in media from *O. sativa* by adjusting nutrients and growth regulators. The induction, proliferation, and regeneration of calluses on *O. sativa* are laborious and time-consuming.^70^ As a result, there is a great need to create a universal medium that can be used by many different varieties of *O. glaberrima*.

## 3. Plant Regeneration: A Constraint in Using Biotechnological Tools for Improving *O. Glaberrima*

The smallest genome of all cereals,^71^ rice is the first fully sequenced agriculturally relevant crop. Additionally, *O. glaberrima*’s genome assembly and annotation have already been reported,^72^ opening the door to the possibility of fully utilizing several significant genes in this species. Although nucleus-cytoplasm interactions and various nuclear gene interactions cause a sterility barrier to exist between *O. sativa* and *O. glaberrima*,^48^ recombinant DNA technology has been used as an alternative method to conventional breeding to transfer novel genes across taxonomic boundaries.^73^ Working with living cells and their molecules is essential to biotechnology, and this can be accomplished using a variety of techniques, including molecular breeding, marker-assisted selection, mutation breeding, tissue culture, micropropagation, gene editing, and molecular diagnostic tools.^74^ Since genetic engineering is the most effective method for improving rice and can introduce useful genes into rice with little disruption to the rice genome,^75^ it will be the main topic of this review, along with some aspects of gene editing.

Despite the strong opposition from the anti-biotechnology lobby in Africa, local farmers have already benefited from tissue culture technologies for crops like pyrethrum, sugar cane, cassava, and bananas.^76^ African rice cultivars known as NERICAs were created using embryo rescue techniques and interspecific crosses of *O. sativa* and *O. glaberrima*.^77^ *O. sativa* gene transformation has been regularly carried out in some labs, though it has been indicated that the *indica* rice system is more complicated.^78^ The introduction of foreign genes into plant cells and the subsequent regeneration of the transformed cells are two essential steps in the transformation of plants. The accessibility of gene transfer, which is unsuitable for plant regeneration, has made it difficult to recover transgenic species of many species.^57^ Model varieties like Kasalath (*indica*) and Nipponbare (*japonica*) have established efficient rice transformation protocols using mature as well as immature seeds^79,80^; however, the difficulties associated with sterilization and isolation of immature embryos continue to be a serious barrier for use of these explants in transformation.^81^ In addition, a lot of popular food-producing varieties, like Koshihikari in Japan and IR64 in tropical regions (as well as Suakoko in Nigeria), have been shown to have lower regeneration capacity in mature seed culture systems, making it difficult to effectively improve them through genetic modification.^82,83^ Afolabi et al. were able to develop a regeneration protocol for callus derived from caryopses of native Nigerian rice cultivars.^79^ Although *O. glaberrima* and *O. sativa* are both species that are grown by farmers in Nigeria and other African nations, there is much less information available on callus induction and plantlet regeneration of *O. glaberrima*.

A lack of effective in vitro regeneration systems for local cultivars may be the reason why African rice varieties have not been much better utilized with various genetic modifications.^45,46^ For instance, the callus of cultivar IRGC104165 (*O. glaberrima*)^84^ has recently demonstrated a failure of shoot regeneration. The ability to induce and regenerate calluses is also known to be variety-dependent, as it was demonstrated to prevent the genetic modification of many indica rice varieties.^85^ One of the main obstacles to the genetic improvement of this species may be *O. glaberrima*’s poor regeneration capacity caused by a lack of suitable media. However, since a better transformation protocol for *O. glaberrima* landraces can be developed, this shouldn’t be a barrier to creating good and acceptable varieties. Testing different hormonal concentration gradients (Kinetin, NAA, or others)^55^ can be used to assess regeneration capacity. Additionally, it has been noted that supplementing media with a substance like a coconut water can enhance the transformation and regeneration of both *O. glaberrima* and *O. sativa* landraces.^47,55^ Moreover, it has been demonstrated that *O. glaberrima* has a greater capacity for regeneration when the concentration of sucrose in the regeneration media is increased.^63,66^

## 4. Factors for Improving Plant Regeneration in *O. Glaberrima*

**Figure 1.**
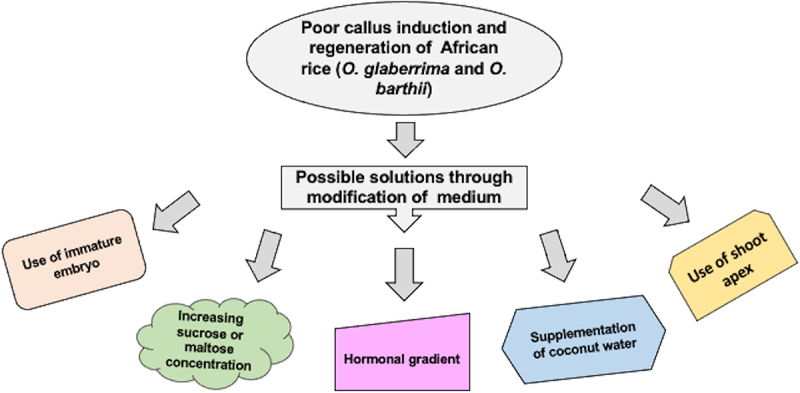
Possible solutions for callus induction and regeneration challenges of African rice *Oryza glaberrima.*

Biotechnologists should first focus more on developing an appropriate transformation and regeneration protocol for *O. glaberrima* to successfully manipulate genetic material and increase rice yield production in Africa. Rather than concentrating on imparting those traits *to O. sativa*, they should focus on improving *O. glaberrima*, which is already adapted to the challenging environmental conditions of Africa. How can we enhance *O. glaberrima*’s transformation and regeneration capacity ? The following strategies may be used to enhance African rice’s capacity for tissue culture (Figure. 1).

### 4.1 Modifying Sucrose or Maltose Concentration of Media

Both embryonic development and somatic embryogenic induction are significantly influenced by the type of carbon source and its various concentrations.^86,87^ Since sucrose produced more biomass than fructose and glucose combined, it is known to be the most effective carbon source for causing callus.^88^ However, sucrose has a different rate of organogenesis than maltose and may have a higher osmotic potential, which causes callus browning^89^ and, at concentrations of 6% or higher causing necrosis.^90^ Brisibe et al. study showed that the high frequency of shoot induction in both liquid and agar culture occurred at 5% across the range of sucrose concentrations tested (0–15%).^63^

Indeed, by increasing the sucrose concentration of MS media from 3 to 5%,^66^ a recent study by Mmbando et al. was able to regenerate transgenic plants from one African rice cultivar, TOG12380 (*O. glaberrima*). However, only one cultivar of the five *O. glaberrima* tested in that study was regenerable. Maltose, on the other hand, has demonstrated superiority for green shoot regeneration in barley^91^ when compared to sucrose as a carbon source for androgenesis. According to Sah et al.,^92^ 4% maltose resulted in the highest levels of callus induction (90.33%) and regeneration (82.66%) of the Japanese rice variety Kitaake. In addition, the number of days to callus formation in the media was reduced by maltose (4%) rather than sucrose.^92^ To create the best tissue culture media for this species, we may need to modify and test different maltose concentrations in *O. glaberrima*. Although Mmbando et al.^66^ were able to regenerate *O. glaberrima* at 5% sucrose, the small number of transgenic lines found in this study may be a result of the high sucrose concentration’s impact on the regeneration media. Agar concentration changes can also affect the induction and regeneration of calluses.^93^ Future research should therefore examine the extent of *O. glaberrima* callus induction and regeneration at 4% maltose as well as by varying the type and concentration of agar.

### 4.2 *Hormonal Gradient for Determining Optimum Condition for* O. Glaberrima

The induction and growth of calluses are known to be significantly influenced by auxin.^94^ Additionally, it has been demonstrated that combining different auxin types is a superior alternative to using a single auxin for the callus induction of rice.^95^ For instance, a hormonal gradient was created based on the optimal concentration of most *O. glaberrima* accessions, which has been shown to be 0.5–4 mg L^–1^ kinetin and 0.01–0.05 mg L^–1^ α-naphthaleneacetic acid (NAA).^55^ The best hormonal setting for NERICA^96^ regeneration has been demonstrated to be 1.3 µM of NAA and 50 µM of kinetic. Other studies suggested only using 11.16 µM of benzylaminopurine (BAA) for *O. glaberrima* regeneration.^46^ Therefore, the success of tissue culture and, consequently, genetic modification *of O. glaberrima* depends on the combination of these growth hormones. However, *O. sativa* is the basis for the majority of the established ideal hormonal conditions, and *O. glaberrima* has no suitable media.^47^ The best conditions for *O. glaberrima* callus induction and regeneration can be discovered by performing a hormonal gradient. Through the use of hormone gradient plates and the addition of coconut water to the growth media, Lacchini et al.^55^ revealed a wide variation in the ability of 13 different African rice accessions to regenerate. In addition, based on earlier studies,^66^ Mmbando et al. tested various hormonal concentrations. They discovered that cultivar TOG12380 (*O. glaberrima*) could regenerate when Brisibe et al. (1990)^63^ media were given a sucrose concentration increase to 50 g L^−1^ The MS medium with 10 µM 2,4-D was deemed suitable for *O. glaberrima* callus induction despite the lack of a hormonal gradient in that study, while the sucrose concentration was increased to 50 g L^−1^ with 0.1 µM of 2,4-D and 50 µM kinetin for regeneration. In the future, it will need to be determined whether using maltose rather than sucrose with the same hormonal combination will produce better results. Additionally, adding a hormonal gradient to the medium, as suggested by Mmbando et al.,^66^ may improve *O. glaberrima*’s ability to grow in tissue culture.

### 4.3 Supplementation of Coconut Water in Callus Induction Media

A routine tissue culture system that includes callus induction and plantlet regeneration is a basic requirement for the genetic modification and generation of transgenic rice plants. Supplementing with coconut water is one way to ensure that your calluses are healthy (CW). Rice,^47,97^ and even other species^98^ have demonstrated that CW, the colorless liquid endosperm of green coconuts (*Cocos nucifera*),^99^ increases the size of the calli and regeneration. For example, Mamun et al. (2004) showed that 2, 4-D, and 10% coconut milk produced the highest amount of regenerative callus from leaf sheath in sugarcane.^100^ Because coconut water contains more sugars^101^ and is rich in essential amino acids like methionine, histidine, lysine, and cysteine,^102^ it has been shown to improve the ability of tissue culture. Additionally, it contains a lot of minerals, such as potassium, vitamins, magnesium, and calcium,^103^ all of which promote rapid callus initiation. Although coconut milk increased callus size in the majority of cultivars, at least some *O. glaberrima* variety has been reported to not require coconut milk for callus formation.^47^ On the other hand, a recent study by Lacchini et al. used coconut water to enhance the medium and regeneration of African landraces.^55^ Since the study was primarily conducted on African rice landraces, it provided an opportunity to design the best media for African rice. The inclusion of CW should be viewed as a crucial element in creating the best media for African rice, *O. glaberrima*.

### 4.4 Use of Immature Embryo Instead of Mature Seeds

Immature embryos may be one way to improve *O. glaberrima*’s capacity for transformation because conventional methods for transforming wild *Oryza* accessions typically fail to produce calli from the scutellum tissue of embryos in mature seeds.^104^ As demonstrated on *indica* varieties that are resistant to transformation and tissue culture,^105,106^ immature embryos have shown to be a successful method for introducing genes without inducing callus.^80,105^ As recently demonstrated on cultivar IRGC104165 (*O. glaberrima*),^84^ most *O. glaberrima* have poor callus induction, which fails shoot regeneration. Thus, introducing the gene of interest by immature embryos rather than mature seeds might increase the regeneration ability of this species. Although Mmbando et al. attempt to transform the *O. barthii* cultivar (TOB7307) using an immature embryo failed, a study by Shimizu et al.^104^ showed that wild *Oryza* could be transformed using modified immature embryo techniques. The different samples and transformation and regeneration conditions used in these two studies may be to blame for the dissimilar findings. Additionally, aside from the impact of the Agrobacterium strains used,^80^ heat treatment and centrifugation before *Agrobacterium* infection can increase transformation efficiency in cultivated maize, wheat, sorghum, and rice.^105,107^ Thus, including these procedures in the *O. glaberrima* embryo at an early stage could improve transformation effectiveness, enable genome editing, and hasten research on other wild *Oryz*a genetic resources. In a recent study, Liang et al. improved *Agrobacterium*-mediated transformation and regeneration methods for *indica* rice from immature embryos as explants by disrupting SUB1A as a test case via CRISPR-Cas9.^108^ Since *O. glaberrima* and *indica* are both known to be resistant to tissue culture, it may be worthwhile to modify the medium for Ciherang-Sub1 and test it with *O. glaberrima*. Although there are a few exceptions,^55,66,109^ *O. glaberrima* varieties^45,46,63,84^ frequently fail to transform using the mature embryo method; therefore, the immature embryo method may be used on this species. We must not ignore the fact that, despite being a good alternative, this protocol is expensive and necessitates more time and labor to dissect immature embryos.

### 4. 5 Use of Rice Shoot Apex (Meristem) in Transformation and Regeneration

The ability to induce and regenerate calluses is genotype-dependent, which sets limits on the genetic engineering of *indica* and African rice varieties. The main obstacles to using these explants in transformation include multiple stages of subculture to select the transformed calli, loss of regeneration potential in calli, availability of immature embryos only during a specific timeframe, and difficulties with isolation and sterilization of immature embryos.^81^ The main benefit of shoot meristem-based multiplication systems, however, is that they allow for the quick and direct production of transgenic plants from transformed shoot apices^70^ and are typically genotype-independent, making them applicable to various genotypes.^81^ As a result, using the rice shoot apex for transformation and recovering *O. glaberrima* through *Agrobacterium*-mediated transformation could be a potential solution. This approach has proven to be simple to use and capable of preserving cultivar integrity.^110,111^ Changing the growth regulators and carbon source of plants while using the shoot apex may make it possible to create an effective and speedy protocol for *Agrobacterium*-mediated genetic transformation of African rice varieties. The generation of chimeric during regeneration, however, is one of the complications linked to this technique that may impede the development of transgenic plants.^112^

## 5. Enhancing Yield Potential of *O. Glaberrima* through Gene Editing: Target Traits

How to increase food production is a major issue for the development of many African nations. The best way to increase rice production in Africa may therefore be to use modern biotechnology to increase the native African rice variety *O. glaberrima*’s resistance to both biotic and abiotic stresses. Despite negative press in Africa, it has been proposed that biotechnology is a key driver for commercializing crops that provide all with a good standard of living and jobs.^113^ Although the issues and difficulties related to biotechnology in Africa will not be the focus of this section, these topics have already been covered elsewhere.^10,75,76,114–121^ Recently, several gene editing methods based on the transcription-activator^122^ like effector nucleases (TALEN), zinc finger nucleases (ZFNs), CRISPR/Cas9 system, and meganucleases have been developed. Agroinfiltration (true agro-infiltration, agro-inoculation or infection, and floral dip), cisgenesis, and grafting (non-genetically modified (GM) scion on GM rootstock or the opposite) are additional new plant breeding techniques (NPBT). The design of a guide RNA (gRNA) for CRISPR genome editing has proven to be simpler than that for TALENs and ZFNs.^54^ The safety of GM crops like rice for human consumption is a major concern, particularly the health risks that could arise from consuming GM foods that contain toxins and allergens.^123^ With the complexity of the biosafety regulation being reduced,^54,124^ recent gene editing techniques utilizing the CRISPR/Cas 9 protocol have demonstrated an improvement in simplicity, amicability, precision, adaptability, and efficiency in the process of genetically modified crops. Additionally, it is simpler to implement and calls for creating a guide gRNA of roughly 20 nucleotides that is complementary to the DNA stretch within the target gene that results in the production of accurate insertion or deletion events.^54,75^

Because the gene to be modified is already present in the plant’s DNA,^125^ CRISPR/Cas 9 edited crops have additional advantages over transgenic plants as opposed to the transgenic method, which produced random insertions and phenotypes. Particularly in Africa, where there are many opponents of using GM crops and biotechnology, CRISPR/Cas9 mediated genome editing (CMGE) may present a great opportunity for editing the undesirable traits of *O. glaberrima* with a significant reduction in problems resulting from GM crops. Simple procedures in CMGE make it possible for even a small laboratory with a basic plant transformation setup typically for many lower-income countries in Africa and other developing countries to carry out genome editing projects.^54^ To carry out the CRISP-Cas9 project, the following steps must be taken: first, locate the protospacer adjacent motif (PAM) sequence in the target gene; second, create a single gRNA (sgRNA); third, clone the sgRNA into an appropriate binary vector; fourth, introduce the sgRNA into host species/cell lines by transformation; fifth, screen; and finally, validate the edited lines.^54^ Although the CMGE method has made it possible to create non-genetically modified (Non-GMO) crops with the desired trait and increased yield under a variety of abiotic and biotic stress conditions, it has been much less utilized to increase *O. glaberrima*’s yield production. The creation of significant off-target cleavage sites has been one of some CRISPR/Cas9 systems’ drawbacks. However, among the suggestions for minimizing this issue are the lengthening of the protospacer adjacent motif^54^ and the use of a more precise gRNAs design approach.^126^ But the question is, can Africa benefit from these new gene editing techniques? Can these techniques enhance *O. glaberrima*’s nutritional value and yield, which is underutilized but important for both local African farmers and the rest of the world? In this study, several methods are suggested for removing all undesirable traits from *O. glaberrima* by CMGE.

This study suggested that the CMGE technique could be used to increase the yield of *O. glaberrima* and even accelerate its domestication,^55,127–132^ similar to the increase in yield in *O. sativa* caused by the simultaneous introduction of three trait-related quantitative trait loci (QTLs)^133^ Since the publication of the *O. glaberrima* genome in 2014,^72^ genes for numerous agronomic traits in rice, including *GRAIN WEIGHT 2* (*GW2), GRAIN NUMBER 1A* (*GN1A*), and *GRAIN SIZE 3* (*GS3*), have already been reported.^134^ By stacking numerous unfavorable traits among this species, it opens the door for yield improvements in *O. glaberrima*.^55,135^ *O. glaberrima*’s main drawbacks^136^ include lodging, low yield, grain shattering,^8^ and possibly UVB sensitivity.^17^ The inexpensive and straightforward method of CRISPR/Cas9 may provide a great opportunity to increase the yield of this species by carrying out multiple knockouts of the genes encoding for these traits in *O. glaberrima*. Instead of being the sole genetic resource for improving *O. sativa*,^18^ which cannot survive in Africa’s harsh environment and results in low production for local farmers who depend on rice as a source of income, this will quicken its breeding programs. A further method for increasing the yield of *O. glaberima* might involve editing the yield genes grain number *1a* (*Gn1a*) and *DENSE AND ERECT PANICLE1* (DEP1), which are important contributors to rice yield increases. Genes like OsGRAS19 and D26,^137^ which control *O. sativa* grain size as well, have the added benefit of enhancing the grain quality of this species. Additionally, the high UVB sensitivity of native African landraces could be reduced by inhibiting the downstream molecular pathways, such as growth-regulatory factors, microRNA396 or hormone-associated genes^138,139^ or other transcription factors, that cause UVB-induced leaf growth inhibition of cell proliferation.

Since the genes that act as a barrier to interspecific hybridization for breeding programs of the two species have already been identified,^50,51,140^ it is possible to use the CRISPR/Cas9 technique to eliminate these genes in *O. glaberrima*. We cannot, however, rule out the possibility that *O. glaberrima* would not survive in the harsh environment of Africa if all of these genes were removed. Many essential genes must be overexpressed for many agriculturally significant traits, such as increased photosynthesis, to occur, as their knockout causes seedling lethality.^141^ Therefore, before approving modified plants, field trials evaluating the efficacy and lethality of these multiple knockout-generated mutants should be carefully assessed. The CRISPR/Cas9 technique has also been used to induce resistance to bacterial leaf blight (BLB), one of the most harmful vascular diseases of rice; which is caused by the proteobacterium *Xanthomonas oryzae* pv. oryzae (*Xoo*) and causes 75% of yield loss with millions of hectares annually.^142^ The sugar-transporting SWEET gene family in rice is typically the target of transcription activator-like effectors (TALEs), which are primarily in charge of releasing the sugar into the apoplast for the nutrition of the *Xoo* pathogens.^143^ For instance, using the CRISPR/Cas9 technique, the introduction of InDels into the effector-binding element (EBE) of the OsSWEET13 promoter of the MS14K line and the creation of a novel rice line with three edited OsSWEET EBEs led to broad-spectrum resistance against all of the tested *Xoo* strains.^144^ Additionally, Olivia et al.^145^ introduced mutations at the promoters of all three SWEET genes at EBEs recognized by different *Xoo* TALEs using CRISPR/Cas9. This resulted in strong and widespread BLB resistance. A CRISPR/Cas9 targeted mutation in the ethylene-responsive factor, OsERF922 in rice,^146^ significantly increased the resistance to blast disease caused by *Magnaporthe oryzae*.

Similar results have been seen when the MLO allele has been eliminated in tomato and wheat.^147,148^ Even though the majority of *O. glaberrima* varieties have demonstrated resistance to certain diseases, such as the rice yellow mottle virus,^36,37^ the CMGE technique may still be able to help the susceptible lines increase some biotic resistance, as it has been done with other crops.^149,150^ Furthermore, *O. glaberrima* genes have been used to enhance particular grain quality traits in high-yielding *O. sativa* hybrid cultivars.^43^ The development of strategies for the increased milling and cooking of African rice has been made easier by QTL mapping grain quality traits, particularly *hr3, Amy6*, and *Alk6-1* from an interspecific cross between *Oryza sativa* and *Oryza glaberrima*.^42^

Through non-host gene insertion in *O. sativa*, transgenic rice with a variety of agronomically significant traits, such as seed storage protein Bean -phaseolin,^151^ pea legumin,^152^ soybean glycinin,^153^ and daffodil phytoene synthase, provitamin A,^154^ has already been produced. These traits offer opportunities to improve the nutritional^155,156^ quality of *O. glaberrima* by CMGE. Because *O. sativa* and *O. glaberrima* are both growing in SSA, increasing the production of both species using CRISPR/Cas9 techniques as well as genetic engineering is crucial for feeding the world’s expanding population, especially Africa, which is the hotspot. Since rice is used as both a food and a cash crop, increasing rice production will also help poor farmers’ economies.

## 6. Conclusion

African rice varieties^45,46,63,84^ and *indica*^105,106^ are both known to be resistant to tissue culture and transformation, which impedes genetic modification methods. Therefore, it is essential to develop a suitable transformation and regeneration protocol to unlock all of these species’ advantageous traits, which could significantly improve food security. The highly complex process of biotechnology necessitates a certain critical mass of technical, financial, and intellectual resources. African governments must educate their people about the advantages of biotechnology for agriculture, industry, and health care, and develop strong policies to support emerging gene-editing technologies. The development of global regulatory frameworks for gene-edited crops is necessary before the implementation of CRISPR technology for food crops, particularly in Africa, where there are many anti-biotechnologists.^75,76,115,118,119^ Due to the importance of these molecular biology techniques, African governments must give science a higher priority in their budgets. To do this, they must train more biotechnologists and set up contemporary labs with sufficient molecular biology reagents and equipment. The pyramiding of genes for the improvement of *O. glaberrima* can also make use of the abundance of rice sequencing data and the accessibility of numerous QTL markers. On the other hand, African researchers and scientists need to take an interest in enhancing *O. glaberrima* and form partnerships with universities and biotech companies in developed countries. It is now time for Africa to develop the skills necessary to feed her population using her resources, including professionals and crops like rice (*O. glaberrima*). The mind-set of the majority of African scientists must be changed in order for this to be accomplished. *O. glaberrima* is still grown in many regions of SSA by local farmers.^9^ Local farmers still prefer *O. glaberrima* over many improved *O. sativa* and NERICA varieties,^10^ which shows that breeders need to pay more attention to this underappreciated but crucial species for SSA. Local farmers in SSA have the chance to increase the yield and nutritional value of *O. glaberrima* by using contemporary genetic editing tools like CRISPR-Cas9. Therefore, this study put forth the hypothesis that genome editing techniques may be an ideal tool for eradicating those undesirable traits through the insertion of non-host genes, regulation of transcription or translation, and modification of host sequences, rather than ignoring this species with economically significant traits for poor farmers in SSA. Future research should investigate whether altering these genes in *O. glaberrima* could affect the organism’s resistance to additional stresses on the continent of Africa.

This study highlighted the advantages and disadvantages of *O. glaberrima* relative to *O. sativa* and the necessity of enhancing this species for high yield production in SSA. This study also offers some recommendations for improving *O. glaberrima*’s capacity for regeneration. To fully utilize the benefits of molecular breeding techniques on this species, this study urged the urgent development of an effective transformation and regeneration protocol for *O. glaberrima* and other African landraces. This study offers suggestions for enhancing *O. glaberrima* breeding and yield to feed the expanding global population, particularly in Africa.

Possible ways for having high transformation and regeneration in African rice varieties
